# Leriche syndrome in a patient with acute pulmonary embolism and acute myocardial infarction: a case report and review of literature

**DOI:** 10.1186/s12872-019-01288-0

**Published:** 2020-01-17

**Authors:** Xuanqi An, Rui Fu, Zhihui Zhao, Xinhai Ni, Changming Xiong, Xiansheng Cheng, Zhihong Liu

**Affiliations:** State Key Laboratory of Cardiovascular Disease, Center for Pulmonary Vascular Diseases, Fuwai Hospital, National Center for Cardiovascular Diseases, Chinese Academy of Medical Sciences and Peking Union Medical College, No. 167 Beilishi Rd, Xicheng District, Beijing, 100037 People’s Republic of China

**Keywords:** Acute pulmonary embolism, Acute myocardial infarction, Leriche syndrome, Aortoiliac occlusive disease

## Abstract

**Background:**

Both acute myocardial infarction and acute pulmonary embolism are distinct medical urgencies while they may conincide. Leriche’s syndrome is a relatively rare aortoiliac occlusive disease characterized by claudication, decreased femoral pulses, and impotence. We present the first case of concomitant acute pulmonary embolism, acute myocardial infarction, and Leriche syndrome.

**Case presentation:**

A 56-year-old male with a history of intermittent claudication was admitted for evaluating the sudden onset of chest pain. Elevated serum troponin level, sustained high D-dimer level, ST-T wave changes on electrocardiogram, and segmental wall motion abnormality of the left ventricle on transthoracic echocardiography were noted. Pulmonary Computed Tomography Angiogram revealed multiple acute emboli. Aortic Computed Tomography Angiogram spotted complete obstructions of the subrenal aorta and bilateral common iliac arteries with collateral circulation, maintaining the vascularization of internal and external iliac arteries. We stated the diagnosis of acute pulmonary embolism and Leriche syndrome and initiated oral anticoagulation. However, Q waves on electrocardiogram and wall motion abnormality on echocardiography persisted after embolus dissolved successfully. Coronary computed tomography angiogram found coronary arterial plaques while myocardial Positron Emission Tomography detected decreased viable myocardium of the left ventricle. We subsequently ratified the diagnosis of concurrent acute pulmonary embolism, acute myocardial infarction, and Leriche syndrome. The patient was discharged and has been followed up at our center.

**Conclusion:**

We described the first concurrence of acute pulmonary embolism, acute myocardial infarction, and Leriche syndrome.

## Background

Both acute pulmonary embolism (APE) and acute myocardial infarction (AMI) are medical emergencies with high mortality rates [1]. Recognizing one from the other could be challenging because of their similar clinical manifestations [2]. Moreover, they sometimes co-occur [3, 4]. Leriche syndrome, also known as Aortoiliac Occlusive Disease, is a relatively rare clinical condition characterized by atherothrombotic obliteration of the infrarenal aorta and both common iliac arteries [5–7]. Here we report a case of concomitant AMI, APE and Leriche syndrome. Pertinent articles have also been reviewed to explore their underlying mechanisms.

## Case presentation

Our patient was a 56-year-old male with a history of hypertension, dyslipidemia, gastric ulcer and long-time smoking. He was admitted to a local hospital’s emergency department with abrupt onset of excruciating substernal pain, dyspnea, and diaphoresis at midnight. His serum troponin I level was 2.4 ng/ml (< 0.04 ng/ml). The initial electrocardiogram (ECG) showed sinus rhythm with horizontal ST-segment depression in leads V2 to V5 while his previous ECG 1 year ago was normal. Transthoracic Echocardiography (TTE) revealed hypokinesia in the posterior-inferior wall of the left ventricle. Local physicians diagnosed AMI presumptively and introduced oral medications, including dual antiplatelet, beta-blockers, and statin. However, the patient unfortunately developed ventricular fibrillation before coronary catheterization and became unconscious. After 30 minutes’ successful resuscitation, markedly decreased platelet level of 18*10^^9^/l (100–400*10^^9^/l) and significantly elevated D-dimer of 43,460 ng/ml (< 2000 ng/ml) were noted. His second ECG discovered new ST-segment elevation in Lead II, III, and aVF. The physicians in the local hospital did not consider him suitable for coronary catheterization for fear of bleeding and commenced platelet transfusions. His symptoms resolved in 5 days, and he was subsequently referred to our center for further evaluation. He was also noted to have a history of intermittent claudication accompanied by occasional amaurosis for 13 years without erectile dysfunction. Furthermore, he complained of transient left calf pain during the initial chest pain episode. Family history did not reveal anything significant.

**Fig. 1:**
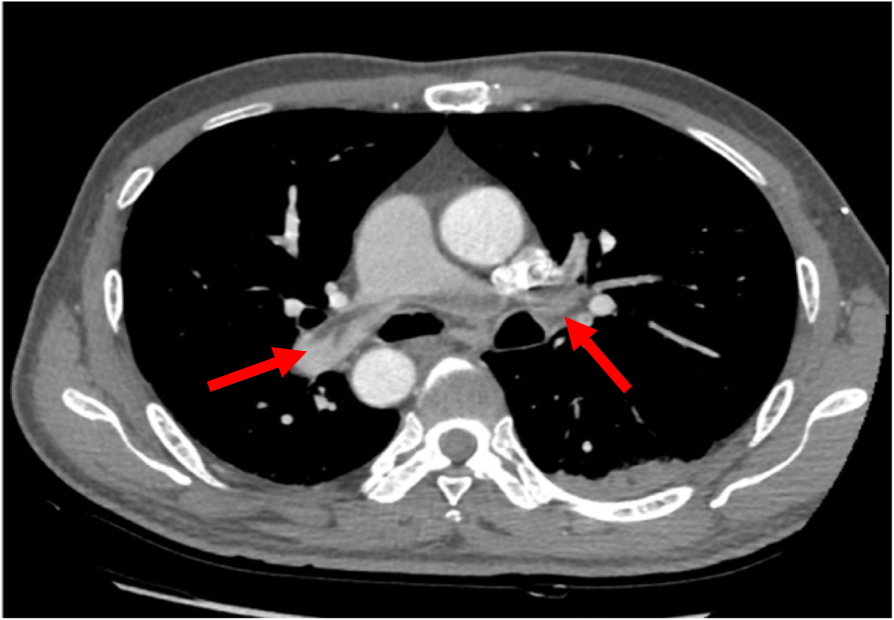
Computed tomography angiogram of the entire aorta showed multiple newly-formed embolus in pulmonary arteries including main branches (indicated by red arrows)

**Fig. 2:**
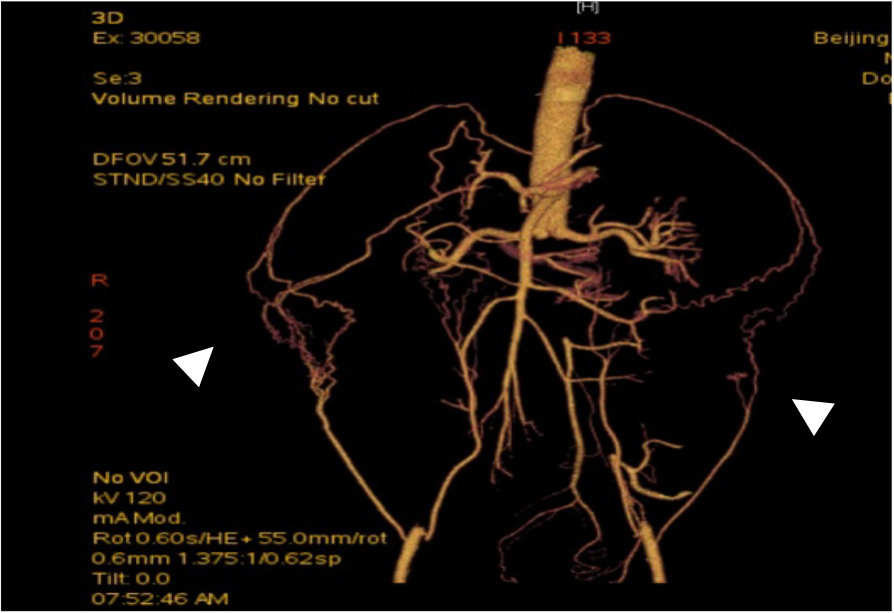
Computed tomography angiogram of the entire aorta showed diffuse calcified occlusions affecting infra-renal abdominal aorta to bilateral common iliac arteries and establishments of collateral vessels (indicated by white arrow heads)

**Fig. 3:**
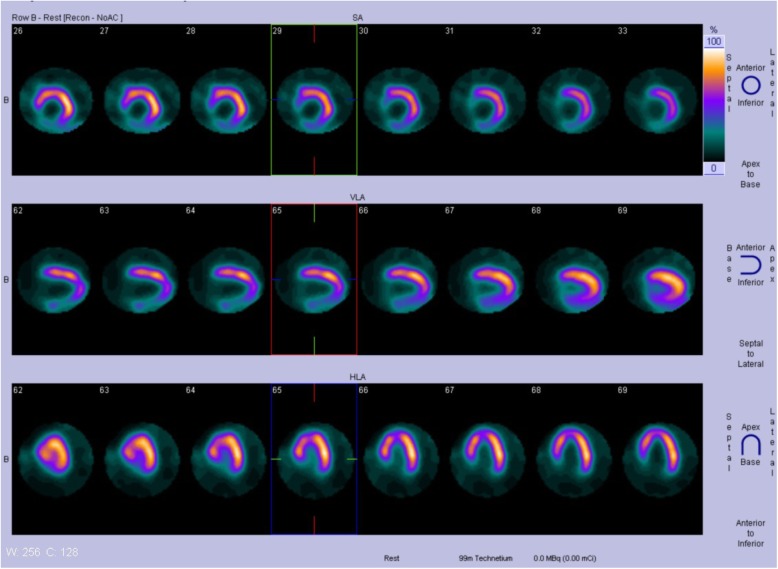
Myocardial perfusion Positron Emission Tomography detected decreased viable myocardium in the inferior wall of left ventricle after successful anticoagulation, The datasets analyzed during the current study are not publicly available due to the fact it is a case report

On admission, his height was 183 cm, and his weight was 80 kg with a body mass index of 23.89. Blood pressure in the upper extremities was 110/60 mmHg, while the number in the lower extremities could not be interpreted. He had a regular heart rate of 83 beats per minute. Oxygen saturation was 96% on room air. Physical examination was remarkable for absent pulsations of bilateral dorsalis pedis arteries. Blood panel showed significantly elevated D-dimer of more than 20.00μg/ml (< 0.5μg/ml), high erythrocyte sedimentation rate of 51 mm/h (< 20 ml/h) and raised sensitivity C reactive protein of 10.87 mg/L (< 5 mg/L). Cardiac enzymes were unremarkable. Lupus anticoagulant increased slightly while antinuclear antibodies and antineutrophil cytoplasmic antibodies remained negative. ECG recorded Q wave in Lead III, aVF, and V7-V9. TTE showed the left ventricle’s ejection fraction of 52% and non-distended left ventricle with posterior-inferior wall hypokinesia. His bilateral Ankle-Brachial-Index were 0, while lower limb arterial ultrasound revealed only plaques and decreased blood flow. With sharp suspicion of pulmonary embolism or aortic dissection, we conducted Computed Tomography Angiogram (CTA) of the entire aorta: multiple newly-formed emboli scattered in main and distal branches of pulmonary arteries (Fig. 1). Diffuse occlusions affecting infrarenal abdominal aorta and bilateral common iliac arteries together with collateral vessels were also noted (Fig. 2). The diagnosis of APE was established based on pulmonary CTA’s findings. His intermittent claudication symptom could be attributed to the diffuse vascular occlusions, which strongly suggested the diagnosis of aortoiliac occlusive disease (Leriche syndrome) [8, 9]. Rivaroxaban 15 mg were given twice per day as anticoagulation. Secondary prevention of coronary heart disease was also implemented, including Aspirin, Clopidogrel, Atorvastatin, and Metoprolol. His D-dimer level resumed within normal range after 17 days. Neither pulmonary CTA nor radionuclide pulmonary perfusion imaging disclosed any thrombus left after 21 days’ treatment. However, Q waves and negative T waves in the Lead III, aVF and V7-V9 persisted on ECG. Hypokinesia in the inferior and posterior walls also remained on echocardiography. Coronary CTA found only calcification and plaques without significant stenosis, while myocardial perfusion Positron Emission Tomography (PET) detected decreased viable myocardium in the inferior wall of the left ventricle (Fig. 3). Multidisciplinary discussion consented to the diagnosis of concomitant AMI and APE. Anticoagulation regimen continued, and vascular bypass surgery was not regarded as a suitable option at that time since the patient was asymptomatic and still in the acute phase of myocardial infarction. His condition was stable at 6, 12, and 18 months’ follow-up at our outpatient clinic after discharge.

## Discussions and conclusions

Our patient kindly provided us a unique opportunity to study this fascinating combination of clinical disorders. He initially presented with chest pain episode, elevated troponin, and d-dimer levels. Serial ECGs revealed a pattern of transient anterior wall ischemia followed by persisted inferior posterior ST-elevation myocardial infarction (STEMI). Subsequent echocardiography showed hypokinesia in the posterior inferior wall. Pulmonary CTA detected multiple newly-formed emboli while aortic CTA showed diffuse occlusion affecting infrarenal aorta and both common iliac arteries with collateral vessels. After three weeks of’ anticoagulation therapy, his D-dimer level normalized, pulmonary embolus disappeared while abnormal findings on ECG, TTE, and myocardial perfusion PET remained. According to the literature, various ECG manifestations have been well described in the setting of APE, including pulmonary P waves, right axis deviations, S1S2S3, S1Q3T3, T waves inversions, ST depressions and ST elevations [2, 10, 11]. Abnormal echocardiography, such as McConnell sign, have also been noted [10–13]. However, these changes were always transient and could resolve quickly after successful anticoagulation therapy [10, 12, 14]. So we proposed that APE and AMI coincided based on the persisted evidence of left ventricular strain after pulmonary embolus dissolved. We found 8 case reports of the coexistence of APE and AMI by searching Pubmed and Medline database using keywords “pulmonary embolism and myocardial infarction.” Three of them have paradoxical embolism, a disease that happened due to patent foramen ovale (PFO), pulmonary AV shunts, or intracardiac shunts [1]. Its standard diagnostic criteria include three aspects: [1] arterial embolism with no evidence of a source in the left heart or arterial circulation [2]; evidence of an abnormal communication between the right and left circulations; and [3] confirmation of deep venous thrombosis or pulmonary embolism [15]. With the prevalence of 27% in the general population, PFO remains still under-diagnosed and under-reported [3]. In this case, although physical examination, repeated TTE, and a cardiopulmonary exercise stress test showed no signs of abnormal communications, we could not rule out the possibility of PFO because of the low sensitivity of TTE [4, 16]. Moreover, since our patient initially had an episode of thigh pain and lower limb ultrasound showed slow venous blood flow after successful anticoagulation therapy, it is possible that deep venous thrombus formed in lower limbs and caused the thrombosis in both pulmonary and coronary circulation through PFO. Another explanation for concomitant APE and AMI is the preexisting coronary arterial stenosis [3, 17]. Triggered by APE, enhanced catecholaminergic discharge could induce vasospasm, and the activation of coagulation cascade could worsen the preexisted stenosis [3]. In our case, since the coronary CTA showed no significant stenosis, this explanation would not suffice. A third possibility would be the procoagulant state. Cancer, estrogen therapy, and autoimmune diseases such as SLE, antiphospholipid syndrome have been well recognized to be related to pro coagulation through different mechanisms [17]. As an elder male, our patient had been screened, especially for malignancy, and no signs were observed.

Another important aspect of our case is the incidental finding of aortoiliac occlusive disease, so-called Leriche syndrome. First mentioned by Irish anatomist Jones Quain in 1847 and later described by French surgeon Rene Leriche in 1940, Leriche syndrome is a rare variant of atherosclerothrombotic occlusive disease characterized by total occlusion of the abdominal aorta and both iliac arteries [7, 18]. Except for typical exacerbating factors such as hypertension, diabetes mellitus, hyperlipidemia, and smoking, other etiologies including developmental defect of aortic growth, radiation exposure, congenital rubella infection, luetic aortitis, Takayasu arteritis, and retroperitoneal fibrosis have also been postulated [19]. It occurs most frequently in males around five decades of life [6]. The classical triad of clinical symptoms is intermittent claudication, impotence, and weak or absent femoral pulses [7]. Collateral vessels so-called choke vessels, always present along with the slowly-developing occlusions while acute aortic embolus extending from the inferior mesenteric artery to both external iliac arteries, could also happen [20, 21]. Comorbidities such as AMI, dilated cardiomyopathy, ischemic bowel disease, gastric outlet obstruction, chronic obstructive pulmonary disease, chronic renal failure, and malignancy have been reported [7, 18, 22]. The preferred treatment regimen range from oral antiplatelet and lipid-lowering therapy to revascularization procedure, including balloon angioplasty, stenting, or bypass surgery [23, 24]. As far as we know, our case is the first case reporting the coexistence of APE and AMI accompanied by Leriche syndrome, which could be attributed to the overlapping nature of their pathophysiology.

As for our treatment strategies, we first adopted LWMH for anticoagulation based on the presence of low-risk APE and concomitant dual antiplatelet therapy for Leiche syndrome and possible AMI. After thrombus dissolved, we switched to rivaroxaban for anticoagulation while reserving dual antiplatelet for confirmed acute myocardial infarction and Leriche syndrome. Not recognized by current guidelines mostly due to rare occurrences of concomitant APE and AMI, our anticoagulation and dual antiplatelet treatment regimen were indeed supported by individual clinical cases [25–27]. Luckily, our patient responded well to the treatment.

So in all, Acute pulmonary embolism could occur concomitantly with acute myocardial infarction. Failure to identify this combination might lead to disastrous consequences. Leriche syndrome is a rare variant of atherosclerothrombotic occlusive disease characterized by total occlusion of the abdominal aorta and/or both iliac arteries. The coexistence of acute pulmonary embolism, acute myocardial infarction, and Leriche syndrome was reported for the first time.

## Data Availability

Data sharing is not applicable to this article as no datasets were generated or analyzed during the current study.

## Abbreviations

AMI: Acute myocardial infarction
APE: Acute pulmonary embolism
CTA: Computed tomography angiogram
ECG: Electrocardiogram
PET: Positron emission tomography
STEMI: ST-elevation myocardial infarction
TTE: Transthoracic echocardiography
